# Influences of substrate and tissue type on erinacine production and biosynthetic gene expression in *Hericium erinaceus*

**DOI:** 10.1186/s40694-025-00194-9

**Published:** 2025-04-03

**Authors:** Elizabeth Doar, Kyle W. Meyer, Zolton J. Bair, Regan Nally, Steve McNalley, Renee Davis, Chase Beathard

**Affiliations:** 1 Department of Research and Development, Fungi Perfecti, LLC, Olympia, WA USA; 2 Independent Researcher, Shelton, WA USA; 3 Department of Civil and Environmental Engineering, University of Washington, Seattle, WA USA

**Keywords:** *Hericium erinaceus*, Erinacine, Liquid culture, Mycelium, Biosynthetic gene cluster

## Abstract

**Background:**

Lion’s mane (*Hericium erinaceus*) mycelium produces erinacines, a suite of cyathane diterpenoids with established neuroactivities. While *H. erinaceus* fruit body tissue has its own characteristic secondary metabolites, it generally does not produce detectable amounts of erinacines. Substrate composition influences the erinacine content of *H. erinaceus* mycelial cultures, similar to production of secondary metabolites in other fungi. This study explored the relationship between biosynthetic gene expression and erinacine content in *H. erinaceus*, comparing fruit body tissue to mycelial tissue cultured in two liquid media formulations.

**Results:**

In this study, we compared erinacine production in *H. erinaceus* fruit body to mycelial tissue cultivated in two liquid media formulations (Complex and Minimal) by quantifying mRNA transcript levels of the erinacine biosynthetic genes *eriE, eriG, eriI, eriC, eriJ, eriB,* and *eriM* (collectively, *eri* genes) alongside high performance liquid chromatography (HPLC) evaluation of erinacines Q, P, A, and C. We also predicted coding sequences for these seven *eri* genes. The Complex media preparation yielded mycelium with significantly higher erinacine C content, while the Minimal media yielded mycelium with greater erinacine Q content, suggesting an alteration of the biosynthetic pathway related to differences in substrate composition. Despite evident differences in erinacine concentrations, mycelial *eri* gene transcript levels did not differ significantly between the two liquid media preparations. When evaluated by gene expression or compound concentration, erinacine biosynthesis was substantially greater in mycelia compared to fruit body tissue in *H. erinaceus*.

**Conclusions:**

Alongside the absence of detectable erinacines within fruit body samples, *eri* gene transcripts were consistently downregulated in the fruit body compared to the mycelium, particularly at early stages of the biosynthetic pathway. Substrate composition is a critical factor in production of erinacines by *H. erinaceus*, and large differences in mycelial erinacine content can occur without significant differences in expression of *eri* genes. Our data support the hypothesis that production of fungal secondary metabolites can be influenced by tissue type and substrate components, and that the expression of *eri* genes is enriched in the mycelium when compared to the fruit body.

**Supplementary Information:**

The online version contains supplementary material available at 10.1186/s40694-025-00194-9.

## Background

**Fig. 1:**
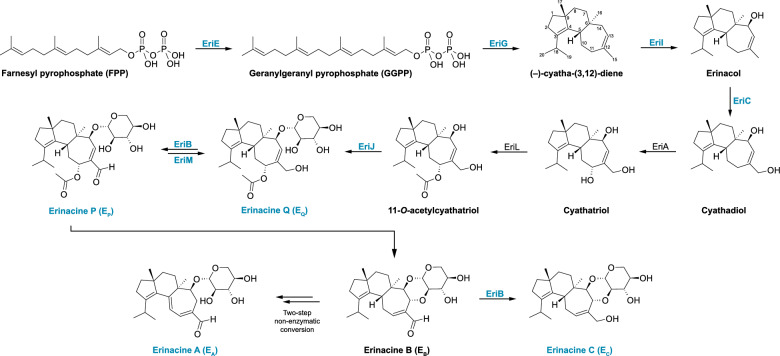
Simplified cyathane diterpenoid biosynthesis pathway highlighting relevant compounds and enzymes. Erinacines and enzymes encoded by genes assessed in this study are labeled in blue

Lion’s mane, *H. erinaceus*, is a fungus valued for its gourmet edible fruit bodies and medicinal benefits. A wide variety of secondary metabolites have been identified in *H. erinaceus* and related species that are associated with myriad bioactivities, ranging from antimicrobial effects to neurogenesis/neuritogenesis [1–3]. *H. erinaceus* preparations have been clinically evaluated for the treatment of depression and anxiety symptoms [4, 5], improvement of cognitive functions [6], mild cognitive impairment [7], and correlates of Alzheimer’s disease in adults [8]. Compounds with established neuroactivities [3, 9–15] produced by *H. erinaceus* include hericenes and hericenones, typically associated with fruit bodies, and cyathane diterpenoids, including erinacines, which are characteristically found in mycelium [16, 17]. Erinacines comprise a diverse suite of compounds biosynthesized in a complex pathway governed by both enzymatic and non-enzymatic reactions. Abbreviating each erinacine as E_X_ (e.g., erinacine A as E_A_), this biosynthetic pathway begins with production of E_Q_ [18] and proceeds from E_P_ to E_B_ to E_C_ or E_A_ (Fig. 1).

The *eri* biosynthetic gene cluster was originally identified as eleven coding sequences localized within 20 kb of a geranylgeranyl diphosphate synthase (GGPPS) gene (Her1A5912/*eriE*, see Additional file 1), designated *eriA* through *eriJ* [19, 20]. This consisted of two UbiA prenyltransferases (*eriF* and *eriG*), a UDP-glycosyltransferase (*eriJ*), three cytochrome P450 proteins (*eriA*, *eriC*, and *eriI*), two NAD(P) oxidoreductases (*eriB* and *eriH*), and one ATP-binding cassette transporter (*eriD*) [20]. Additional genes in the *eri* cluster have since been characterized [21, 22], including three FAD oxidoreductases (*eriK, eriM* and *eriN*), an acetyltransferase (*eriL*), and two more NAD(P) oxidoreductases (*eriO* and *eriP*). While many cyathane diterpenoid compounds and biosynthetic genes have been identified, little is known about the relationship between expression of these genes and erinacine production, particularly in the context of different substrates and types of fungal tissue.

In this study, we explored the impact of liquid media formulations on the expression of genes in the erinacine biosynthetic gene cluster and erinacine content in *H. erinaceus* mycelium and fruit body tissue. We sequenced and characterized the seven *eri* genes highlighted in this study, then used RT-qPCR to quantify *eriE, eriG, eriI, eriC, eriJ, eriB,* and *eriM* mRNA transcripts in *H. erinaceus* mycelium grown in two liquid culture media formulations and fruit body samples grown on a supplemented alder sawdust substrate. After HPLC mass spectrometry (HPLC–MS) was employed to identify E_Q_, E_P_, E_A_ and E_C_, we quantified E_C_ and evaluated levels of E_Q_, E_P_, and E_A_ with HPLC diode array detection (HPLC–DAD).

## Materials and methods

### Sequencing and protein predictions

Sanger sequencing for all genes was performed using the Eurofins Genomics, LLC Tube Sequencing service with PCR products amplified from *H. erinaceus* genomic DNA (gDNA) extracted with a New England BioLabs Monarch Genomic DNA purification kit. Sequencing primers are listed in Additional file 2. With the exception of *eriM* sequencing primers [22], sequencing primers were custom designed with the aid of NCBI’s Primer Blast (https://www.ncbi.nlm.nih.gov/tools/primer-blast/), accounting for primer length, GC content, and Tm.

**Fig. 2:**
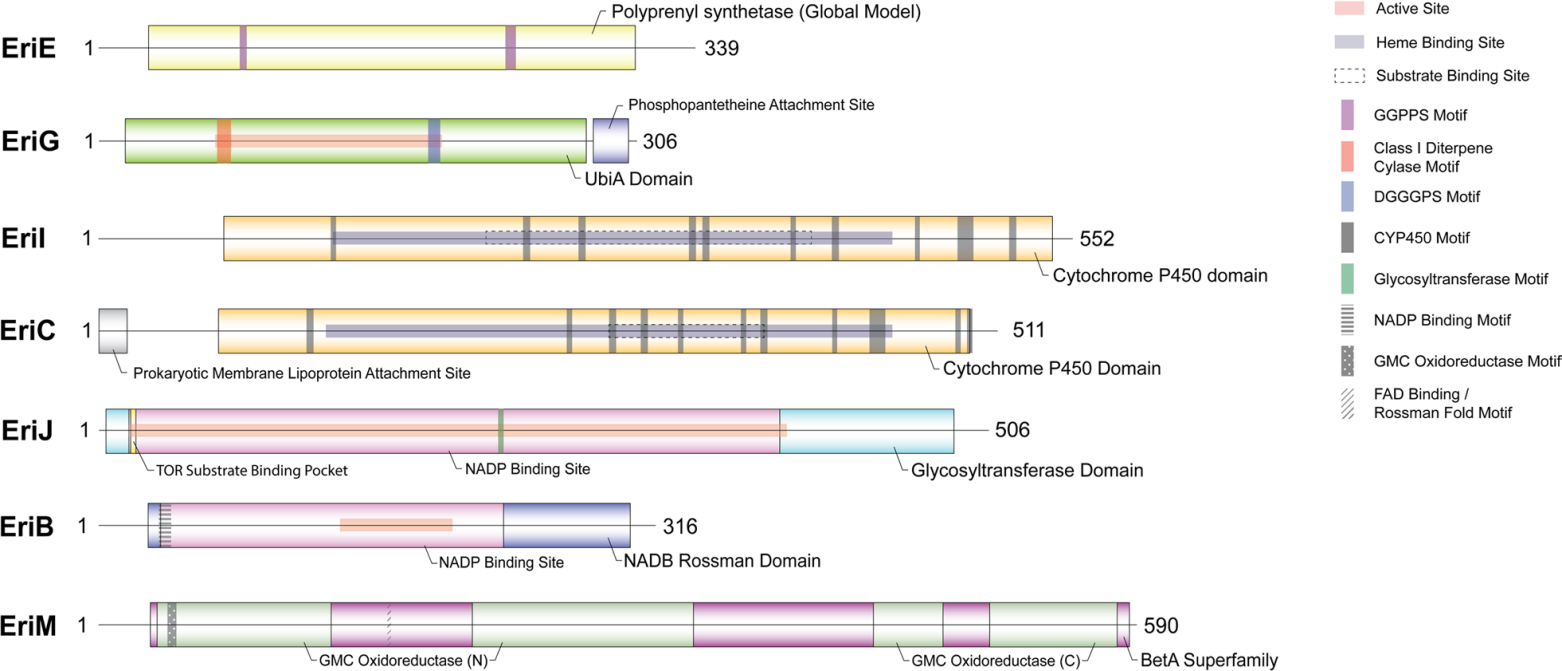
Summary of erinacine biosynthetic enzymes (Additional file 3). Amino acid lengths of each protein are included at the C-termini with predicted conserved domains and motifs represented by boxes along the length of the sequence

Coding sequences were created by aligning gDNA reads with available GenBank *eri* gene coding sequences to determine intron/exon junctions. In the case of *eriM*, the sole gene under investigation without a coding sequence available, the *eriM* gDNA sequence was aligned with *H. erinaceus* transcriptome shotgun assembly GHOV00000000.1 to determine intron–exon junctions and the subsequent coding sequence (PQ561599.1). Resulting protein sequences were scanned for conserved domains and motifs using the NCBI Conserved Domains tool (https://www.ncbi.nlm.nih.gov/Structure/cdd/wrpsb.cgi), Swiss Institute of Bioinformatics (SIB) MyHits (https://myhits.sib.swiss/cgi-bin/motif_scan), Rapid UBIquitination detection (RUBI) (http://old.protein.bio.unipd.it/rubi/), and manual searches. Specific amino acid locations for all predicted protein features are included in Additional file 3. Predictions of key domains (Fig. 2, Additional file 3) were made using the NCBI Conserved Domains tool set to an E value threshold of 0.01, and ubiquitinated lysine predictions provided by RUBI were set to a 99% confidence level. SIB MyHits collects the predictions of several different tools which all have their own methods of evaluation of likelihood; therefore, only the most confident putative motifs (rated as either a strong match, ‘!’, or a strong match for a family-specific motif, ‘!!’) were reported.

### Liquid culture

A mycelial culture of *H. erinaceus* (genetically identified using standard fungal barcoding regions) was sourced from a private culture library and incubated on agar media (40 g/L barley malt extract, 27 g/L agar, 2 g/L yeast extract) in Petri dishes at room temperature for 25 days. To produce a seed culture, three fully colonized agar wedges (approximately 0.5 cm^2^ each) were aseptically transferred from a single plate’s leading edge to inoculate 2 L sterile liquid broth (40 g/L barley malt extract, 2 g/L yeast, 1 g/L CaSO_4_) in a 2.8L Erlenmeyer flask, then stirred at 150 rpm at room temperature and incubated for 21 days. For each liquid culture, 250 mL of broth was prepared in a 500 mL Erlenmeyer flask, with n = 3 Minimal media flasks and n = 3 Complex media flasks. Minimal media was composed of 12 g/L barley malt extract, 0.66 g/L NH_4_NO_3_, and 1 g/L CaSO_4_, suspended in reverse osmosis filtered residential water. Complex media, modified from a previous recipe [23], was composed of 12 g/L dextrose, 5 g/L oatmeal, 1.5 g/L CaCO_3_, 0.5 g/L (NH₄)₂SO₄, and 0.5 g/L lactalbumin enzymatic hydrolysate. CaSO_4_ was ground by mortar and pestle, while oatmeal was homogenized using a handheld coffee grinder and sieved through a Hogentogler & Co., Inc. U.S.A. Standard (ASTM E-11) #20 850 μm test sieve prior to addition to liquid culture flasks. Due to their relative insolubility, CaSO_4_ and oatmeal were weighed and added to each flask separately. Flasks were pressure sterilized at 15 psi for 60 min, and inoculated with 100 mL of seed culture each before incubating in a lidded shaking water bath incubator set to 24 °C and 150 rpm for 21 days.

### Fruit body cultivation and sample preparation

*H. erinaceus* fruit bodies were cultivated on supplemented alder wood sawdust and harvested from colonized blocks. To avoid superficial contaminants, tissue samples were collected for RNA extraction by splitting each fruit body and sampling twice from the center with a sterile scalpel, then immediately flash frozen in epitubes in liquid nitrogen (n = 6). Tissue was stored at −80 °C until RNA extraction. Remaining fruit body tissue was lyophilized at −105 °C and continuous vacuum approaching 0.001 mBar in a Labconco FreeZone 4.5L lyophilizer equipped with an Edwards nXDS 10i dry scroll pump until wholly dried, then homogenized with a Magic Bullet^®^ blender into a fine powder and extracted for HPLC analysis.

### Liquid culture mycelial sample preparation

After a 21 day incubation period, liquid culture tissue was harvested in front of a laminar flow hood. Fungal tissue for RNA extraction was obtained from liquid culture flasks using sterile forceps, immediately flash frozen in epitubes in liquid nitrogen, and stored at −80 °C. The remaining *H. erinaceus* mycelial biomass from each flask was filtered through cheesecloth to separate tissue from liquid media. The resulting biomass from each flask was rinsed with 18.2 MΩ DI water, manually compressed in cheesecloth to remove excess filtrate, then stored in a 50 mL centrifuge tube at −80 °C. Samples were lyophilized in bulk with a collection temperature of −105 °C and under continuous vacuum to 0.001 mBar as described previously. After lyophilization, samples were ground with a mortar and pestle into a fine powder and sampled for HPLC analysis.

### RNA extraction and cDNA synthesis

RNA extraction was performed for each frozen sample separately using a Qiagen RNeasy Plant Mini Kit with an RLT lysis buffer; tissue was first frozen in liquid nitrogen and then homogenized using a pestle in pre-cooled 1.5 mL epitubes. Each RNA stock underwent a DNase I (ThermoFisher, RNase-free, Catalog # EN0521) treatment, which used 4.5 μL of DNase I for every 30 μL of RNA and 30 min of incubation at 37 °C, followed by the addition of 3 μL EDTA and 10 min of incubation at 65 °C to terminate the reaction. The RNA stocks were then reverse transcribed into cDNA using an Applied Biosystems High-Capacity cDNA Reverse Transcription Kit. RNA and cDNA stocks were stored at −80 °C. All RNA stock 260/280 ratios were ≥ 2.0, and all original and diluted cDNA stock 260/280 ratios were ≥ 1.8, as assessed using an Agilent Synergy HTX multimode plate reader. For RT-qPCR, stock solutions of each cDNA sample were created at 100 ng/μL concentrations.

### RT-qPCR and calculations

**Fig. 3:**
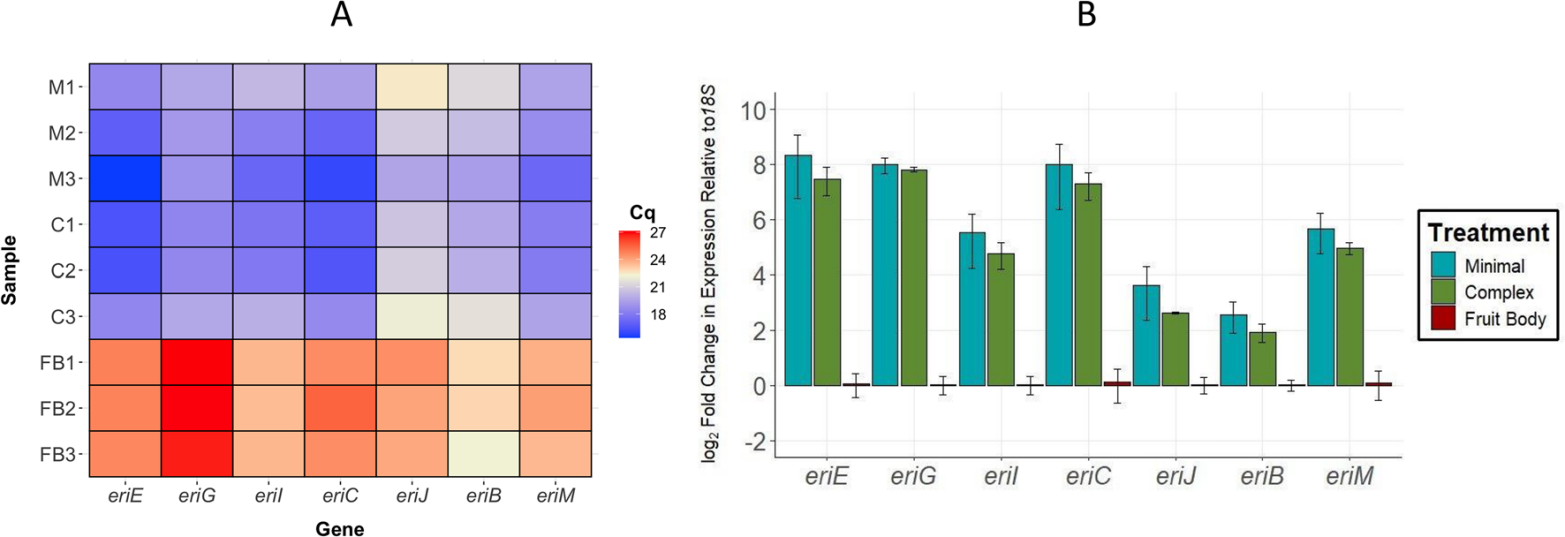
**A** Average Cq values of all *eri* gene primers (n = 4 technical replicates) for mycelia cultured in Minimal media (M) or Complex media (C), as well as fruit body tissue (FB). **B** Fold change in *eri* gene transcript expression relative to *18S* determined by RT-qPCR (n = 3 technical replicates). Results are normalized relative to fruiting body samples. Expression data are represented on a log2 transformed y-axis, with error bars representing the standard deviation of each treatment average

RT-qPCR primer sequences (Additional file 4) were either reproduced [20, 24] or designed originally (*eriE, eriB,* and *eriM*). RT-qPCR was performed with a Rotor-Gene Q system (Qiagen) and used a QuantiNova SYBR Green PCR kit for all reactions. Each reaction used 10 μL of 2X QuantiNova SYBR Green PCR Master Mix, 2 μL of 10 μM forward primer, 2 μL of 10 μM reverse primer, 2 μL of template cDNA at 100 ng/μL, and RNase-free water to 20 μL. Thermocycling conditions were 5 min at 95 °C, then 50 cycles of 5 s at 95 °C, and 10 s at 60 °C. Specificity of PCR products were confirmed using gel electrophoresis; images of gel checks are included as Additional file 5. All RT-qPCR primer pairs amplified bands of the appropriate size in all sample sets, except for the *eriI* primers [20] in fruit body tissue, which also amplified a smaller band below the expected 207 bp band. This issue did not reappear within mycelial tissue checks for these primers, suggesting limited primer specificity amid the low levels of *eriI* mRNA transcript present in fruit body tissue. Actual *eriI* transcript expression (Cq) values gathered from this fruit body material were comparable to Cq values for other primer pairs (Fig. 3A).

Three bioreplicates were prepared for each treatment group: Minimal media, Complex media, and fruit body tissue. The two samples taken from each fruit body mass (total n = 6) were evaluated separately during gel electrophoresis specificity checks and RT-qPCR transcript quantification, and then combined into single bioreplicates (total n = 3) while calculating average Cq values. Average Cq values were calculated using n = 4 technical replicates for *eri* gene primers and n = 2 for *18S* primers. Fold changes in expression were calculated directly from these averages based on the 2^−ΔΔCt^ method [25], using the total fruit body average Cq (Qiagen’s Ct equivalent) as the calibrator sample and *18S* as the reference gene. All statistical comparisons were performed using an unequal variance one-tailed t test, with *p* ≤ 0.05 considered significant.

Two different *18S* RT-qPCR primer pairs, one large subunit (LSU) ribosomal DNA primer pair, and one *β-tubulin* primer pair were evaluated to identify an optimal reference gene for this sample set by comparing mean Cq (n = 2 technical replicates) for all four primer pairs across all three sample sets, determining each sample’s variation from their treatment average Cq value. The *18S* primer pair included in Additional file 4 (and used for all reported Cq values and fold change calculations) was selected because it demonstrated the lowest level of variation across all three sample sets, determined by summing the absolute value of all sample variations for each primer pair and selecting the primer pair with the lowest total sum.

### HPLC sample preparation

Extractions were carried out by combining 250 mg of powdered and lyophilized fungal biomass with 10 mL of a 1:3 solution of MeOH:EtOAc in centrifuge tubes, followed by sonication for 5 min and agitation on a plate vortexer for 30 min. After extraction, each sample was centrifuged for 10 min at 2000 rpm. The supernatant was decanted into a scintillation vial and reduced to residue under pressure with a Buchi R-100 rotary evaporator system. The resulting residue was resuspended in 1 mL of methanol and filtered through a 0.22 µm polypropylene syringe filter prior to analysis by HPLC.

### HPLC–MS and HPLC–DAD analysis

HPLC–MS analyses were carried out by EZ Labs, LLC (San Diego, CA, USA) on an Agilent 1290/1100 series HPLC equipped with an Agilent 6130 series single quadrupole MS detector and UV/PDA detector. HPLC–DAD was performed on an Agilent 1260 Infinity II chromatography system using a diode array detector. Primary detection of analytes (E_A_, E_C_, E_P_, and E_Q_) was at 195, 210, and 345 nm. E_C_ was quantified at a wavelength of 195 nm against an analytical standard originating from material purified by EZ Labs, LLC. A gradient method was employed to achieve separations via an Agilent Poroshell 120 EC-C18 2.7 µm 150 mm × 4.6 mm column. Ultrapure 18.2 MΩ deionized water and HPLC grade acetonitrile were used as A and B components of the mobile phase respectively. The gradient in the method was applied as shown in Table 1. The flow rate was set to 1 mL/min, and column temperature was set to 35 °C.

**Table 1:** Solvent gradient profile for the HPLC method using water (mobile phase A) and acetonitrile (mobile phase B)

Time (min)	% B (Acetonitrile)
0	30
8	41
19	43
22	46
26	48
28	60
32	95
38	95
40	30
44	End

## Results

### Sequencing and in silico modeling of erinacine biosynthetic genes

Our study selected *eriE, eriG, eriI, eriC, eriJ, eriB,* and *eriM* for Sanger sequencing and protein domain prediction prior to an evaluation of mRNA transcript levels. Previously, only six out of seven coding sequences for the *eri* genes selected in this study were publicly available; we predicted the *eriM* coding sequence by aligning its sequenced gDNA region with *H. erinaceus* transcriptome shotgun assembly GHOV00000000.1 to determine intron–exon junctions and obtain a coding sequence (PQ561599.1). Figure 2 summarizes all predicted protein domains and motifs, while more comprehensive results (including tools and confidence levels used) are included as Additional file 3.

### RT-qPCR analysis of eri gene mRNA transcripts

After performing an RT-qPCR evaluation of fruit body tissue and mycelia grown in either Minimal media or Complex media, we compared mean Cq values alongside fold changes relative to the housekeeping gene *18S*. When examining Cq values directly, we found no statistically significant differences in expression of *eri* mRNA transcripts between mycelial tissue cultivated in either liquid medium; however, in all 14 comparisons, *eri* genes were expressed significantly higher in mycelium compared to fruit body tissue (*p* ≤ 0.05; Fig. 3A). Overall, the lowest expression levels (highest Cq values) were observed for *eriG*, *eriC*, and *eriE* in fruit body tissue, and the highest expression levels (lowest Cq values) were observed for *eriE* and *eriC* in mycelia cultured in Minimal and Complex media.

Next, we transformed Cq values into fold changes in expression of *eri* mRNA transcripts by normalizing and reporting all fold changes relative to the fruit body sample set, which had the lowest mean expression for all transcripts (Fig. 3A). Overall, trends in fold changes between treatments and tissue types were highly similar to those observed for unadjusted Cq values. We observed statistically significant differences between fold change values for all seven tested *eri* genes when comparing Complex media mycelium to fruit body (*p* ≤ 0.05), and for three out of seven *eri* genes when comparing Minimal media mycelium to fruit body (*eriG, eriB,* and *eriM*,* p* ≤ 0.05). No statistical difference was observed between Complex media and Minimal media mycelia when comparing fold change values for any of the seven genes examined (Fig. 3B).

### Erinacine production results determined by HPLC–DAD

**Fig. 4:**
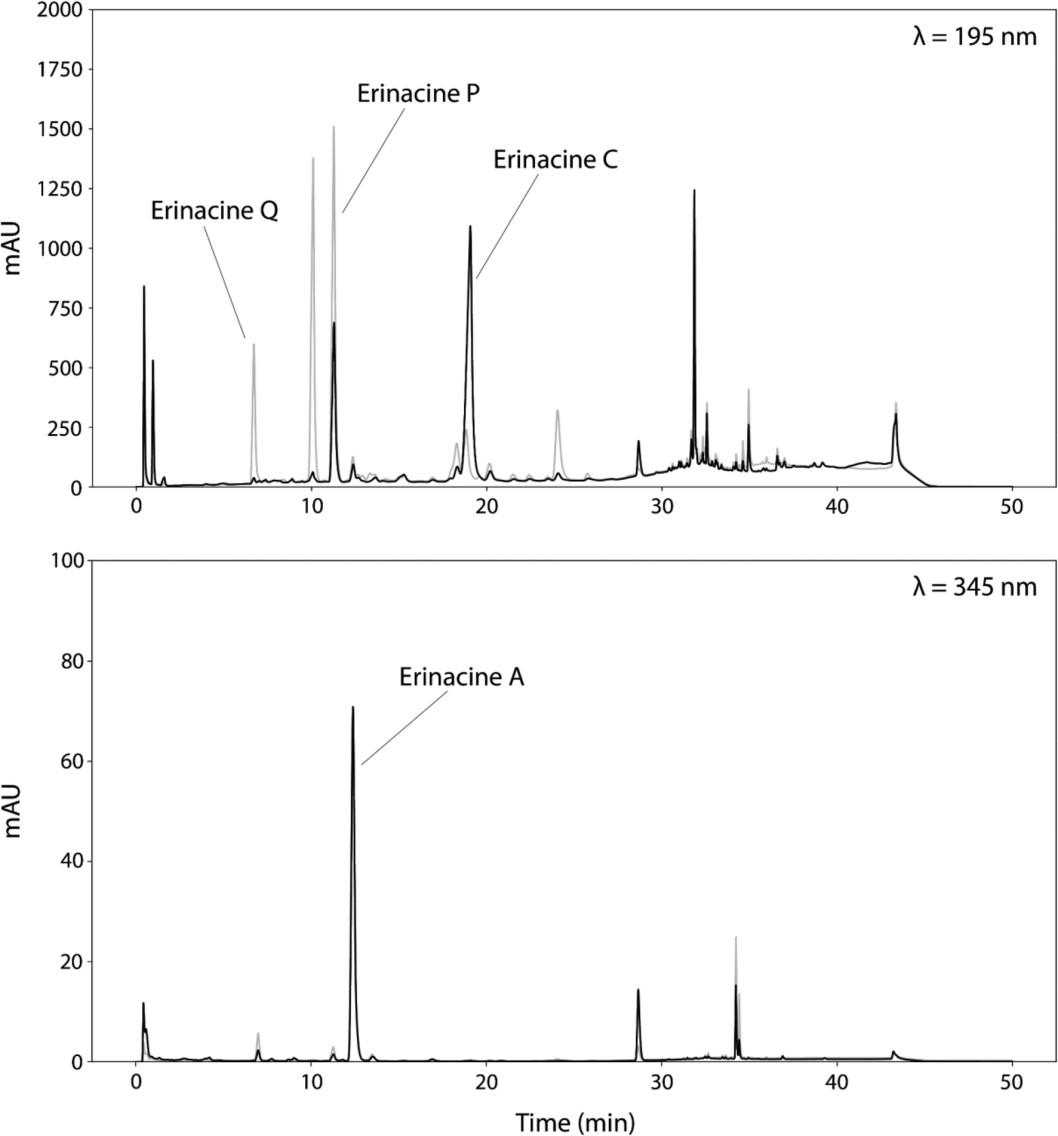
Representative HPLC UV–Vis chromatographic results for single replicates of mycelia cultivated in Minimal media (gray) and Complex media (black) at 195 nm (top) and 345 nm (bottom) detection channels. Minimal and Complex media yielded mycelia that differed in the measured signal intensities of E_Q_ (as determined by integrated peak areas per gram of fungal biomass, AUC/g; Fig. 5B) and the quantified concentrations of E_C_ (Fig. 5A). Samples used for this figure are the highest-yielding flasks for each sample set: Minimal media flask 3 and Complex media flask 2

**Fig. 5:**
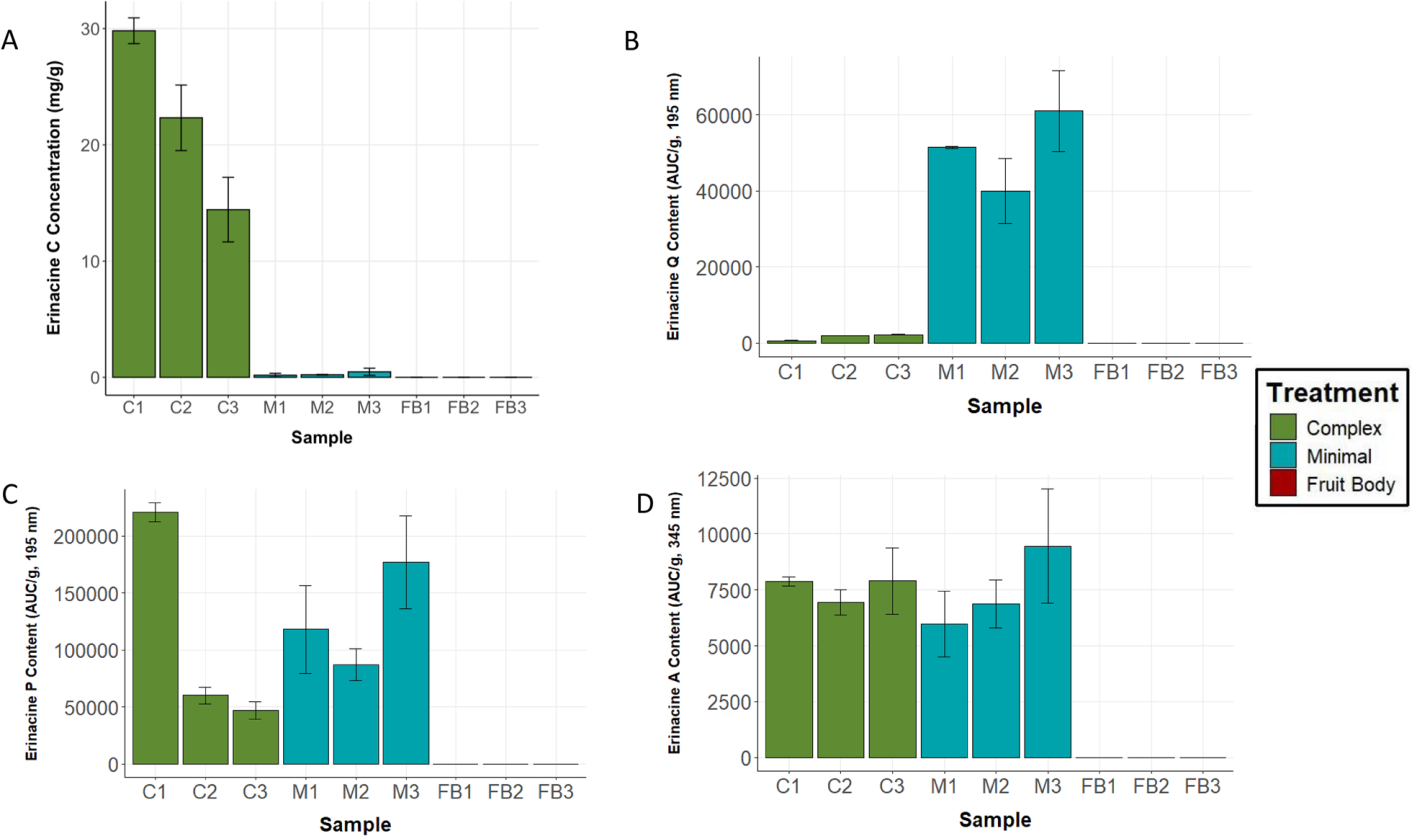
**A** Mean E_C_ content (mg/g dry tissue) as determined by HPLC (n = 3 technical replicates). Error bars represent standard deviations for each flask (each biological replicate tested in triplicate). **B** Average signal response (AUC/g) in dry biomass for E_Q_. **C** Average signal response (AUC/g) in dry biomass for E_P_. **D** Average signal response (AUC/g) in dry biomass for E_A_

A combination of HPLC–DAD and HPLC–MS analyses confirmed the identities of E_Q_, E_P_, E_A_, and E_C_ (Additional file 6). HPLC analysis of mycelia revealed significant variation in E_C_ concentrations, as well as differential production of E_Q_, E_P_, and E_A_, depending on media type (Figs. 4 and 5A–D). E_C_ was detected in mycelium grown in Minimal media, but at concentrations approximately two orders of magnitude lower than concentrations observed in mycelium grown in Complex media (Fig. 5A). Analytical standards of E_Q_, E_P_, and E_A_ were unavailable; therefore, these erinacines could not be quantified directly. However, each compound was individually compared across tissue types using the measured peak area per gram of dry mycelial tissue (AUC/g).

In Minimal media mycelium, a greater diversity of minor erinacines and putative erinacine candidates were observed; E_Q_ displayed the least variation and was present at higher concentrations than in Complex media mycelium (according to relative peak areas). Measured peak areas of E_P_ and E_A_ were statistically indistinguishable between mycelia cultivated in Minimal and Complex media (Fig. 5C, D). In accordance with the higher standard deviations in RT-qPCR results for mycelium grown in Minimal media, signal responses of all erinacines were more variable among replicates in the Minimal media compared to Complex media, evidenced by their relative standard deviations (Fig. 5A–D). Fruit bodies were also evaluated for erinacine content by HPLC, but none of these four erinacines were detected in any fruit body tissue.

## Discussion

According to RT-qPCR results, *H. erinaceus* mycelia demonstrated dramatically higher *eriE, eriG* and *eriC* expression compared to *eriI*, *eriJ*, *eriB*, and *eriM* (Fig. 3B). When evaluating transcript expression patterns, we observed that *eriG* displayed particularly low expression in fruit body, exhibiting the highest mean Cq values of any gene evaluated in either tissue type in this study (Fig. 3A). This may suggest that *H. erinaceus* provides more transcriptional regulation at the beginning of the erinacine biosynthetic pathway, which has parallels with GGPP-based pathways in plants such as carotenoid biosynthesis [26]. Although detectable, there are substantially lower levels of *eri* gene transcripts in the center of fruit body samples compared to mycelial samples; future investigations may be warranted to determine if erinacine biosynthesis could occur in the hymenium. More generally, the significant increase in *eri* gene expression in the mycelium could indicate a wide-ranging lack of engagement of the erinacine biosynthetic pathway in the fruit body. While this does not preclude the possibility of erinacine biosynthesis in fruit body tissue, when combined with our HPLC analyses of these same samples, the low levels of *eri* gene transcripts in fruit body are consistent with the lack of detectable E_Q_, E_P_, E_A_, or E_C_.

The few prior studies performing transcript- and protein-level evaluations of *H. erinaceus* mycelium and fruit body tissue have produced some results inconsistent with our study, potentially driven by differences in detection methods, strains, substrates, and cultivation parameters, as well as time points chosen for sampling. For instance, transcriptome analysis found that *eriA* and *eriC* were upregulated in the fruit body compared to mycelium [19], and a proteomic comparison noted that EriB and EriJ were upregulated in fruit body tissue compared to mycelium [24]. However, these studies both used potato dextrose agar (PDA) media, and their results are also inconsistent with many other studies that have predominantly observed erinacine biosynthesis and presence in mycelium [8, 16, 17, 20, 23, 27], with limited possible exception [28]. Given the well-known influence of substrate composition on the secondary metabolic profile of fungi [29], it is possible that PDA media is not ideal for promoting biosynthesis of erinacines.

In a time-series investigation using a liquid media substrate containing barley malt extract and oatmeal similar to the Complex media used in this study, expression of *eriF, eriG, eriA, eriC, eriI,* and *eriJ* transcripts generally increased until 10 days post inoculation (dpi), with maximum transcript levels concurrent with detection of E_P_ [20]. Our RT-qPCR results at 21 dpi do not align with the elevated levels of *eriC* and *eriJ* noted at 10 dpi by other researchers; in particular, we observed much lower *eriJ* fold change values. Besides timing, another potential source of discrepancy is that this group used *β-tubulin* for normalization of fold change values, while our investigation found *18S* to be less variable in this context. Few studies have investigated precisely when *H. erinaceus* mycelium begins producing erinacines, but E_C_ has been detected as early as 3 dpi and as late as 24 dpi in media similar to Complex media, with maximum mycelial E_C_ content largely plateauing from approximately 6–24 dpi [23]. Additionally, E_P_ has been detected in liquid cultured mycelium as early as 5 dpi [20]. Therefore, substrate and incubation time both have demonstrated potential to influence erinacine production dynamics and have led to varied results. Given that maximum E_C_ content was previously observed to remain steady after 6 dpi [23], liquid-cultured *H. erinaceus* mycelium may have already produced the maximum amount of erinacines possible by 21 dpi, potentially influencing this discrepancy in transcript expression.

Notably, comparisons of both mean Cq and fold changes revealed no statistically significant differences in mRNA transcript levels of any examined *eri* gene when comparing Minimal media mycelium to Complex media mycelium. These RT-qPCR results contrast the distinctly different erinacine production patterns observed. Mycelia grown in each media preparation exhibited comparable amounts of E_P_ and E_A_, but Minimal media mycelia yielded significantly more E_Q_ than Complex media mycelia, and Complex media mycelia vastly more E_C_ than Minimal media mycelia (Fig. 5A–D), suggesting that production of E_C_ in Complex media mycelia may have been favored over other evaluated precursors in the biosynthetic pathway (Fig. 1). One potential reason for this division in erinacine production is the different media formulations. For example, the enhanced production of E_C_ by mycelium grown in Complex media aligns with past observations that increasing the complexity of substrate nitrogen sources can promote production of E_C_ [23]. Similarly, Minimal media includes calcium sulfate dihydrate, while Complex media contains calcium carbonate, which has also been noted as vital to production of E_C_ [23]. Clearly, the nutritional composition of liquid media can have a major effect on the production of secondary metabolites in fungal tissue without necessarily affecting enzyme transcript levels.

Given that the increase in E_Q_ levels within Minimal media mycelium is seemingly at the expense of E_C_, we hypothesize that the erinacine pathway either continues to progress toward production of E_C_ or is largely stalled at preceding erinacines when challenged by nutrient deficiency. Considering their roles in the biosynthetic pathway (Fig. 1), EriL, EriJ, EriB, and EriM are all potential points of enzymatic influence over the secondary metabolite profile of *H. erinaceus*. Furthermore, the overall lack of correlation between levels of *eri* gene transcripts and erinacine content suggests this pathway could also be regulated through post-translational mechanisms. These observations highlight the need for in-depth chemical, enzymatic, and transcript-level time-series studies of erinacine biosynthesis to pursue directed production of specific erinacines within *H. erinaceus.*

## Conclusion

Liquid media composition influences erinacine production by *H. erinaceus* mycelium and may be employed to promote production of specific erinacines. While RT-qPCR evaluation of *eriE, eriG, eriI, eriC, eriJ, eriB*, and *eriM* mRNA transcripts demonstrated strong correlation with patterns of erinacine production in the mycelium compared to the fruit body, they were less predictive of concentrations of specific erinacines in *H. erinaceus* mycelium. Fruit body tissue demonstrated no measurable erinacine content or upregulation of *eri* mRNA transcript levels alongside a notable downregulation of *eriG*, the first committed step of erinacine biosynthesis. To our knowledge, this is the first study to compare the influence of substrate on the production of a suite of erinacines and biosynthetic enzyme transcripts in parallel, and the first to concurrently quantify the difference in production of E_C_ between the mycelium and fruit body. This study highlights the potential for significantly influencing the secondary metabolite profile of a fungus through a change in growth substrate, emphasizing the need for more comprehensive analyses across erinacines produced in response to liquid media formulations. Overall, these results demonstrate that substrate composition can impact production of erinacines in *H. erinaceus* mycelium without significant differences in biosynthetic gene expression, and confirm that several erinacines are abundant in mycelium when compared to fruit body tissue.

## Supplementary Information

**Additional file 1.** Nomenclature for the sixteen genes in the broader *eri* biosynthetic cluster. Identified by Chen et al., Yang et al., and Ma et al.

**Additional file 2.** Sanger sequencing primers. Primers marked with an asterisk were reproduced from Ma et al.

**Additional file 3.** Summary of locations of select predicted protein domains and motifs in EriE, EriG, EriI, EriC, EriJ, EriB, and EriM

**Additional file 4.** RT-qPCR primers. Used to amplify *eri *gene cDNA sequences for RT-qPCR. Primer sequences marked with * were reproduced from Yang et al., and sequences marked with ** were reproduced from Zeng et al.

**Additional file 5.** Gel electrophoresis specificity checks for RT-qPCR primers. See Methods regarding the* eriI *check in fruit body samples.

**Additional file 6.** UV-Vis and mass spectral signatures for erinacines Q, P, A, and C

## Data Availability

The newly available erinacine biosynthetic gene coding sequence is publicly available in the National Institute of Health's database, Genbank, with the accession number PQ561599.1. Raw data for the HPLC data presented in Fig. 5 and Fig. 6 are provided in Additional file 6. All data are available on reasonable request to chase.b@fungi.com.
