# Supplementary material for: Influences of substrate and tissue type on erinacine production and biosynthetic gene expression in Hericium erinaceus
Source: Fungal Biol Biotechnol. 2025 Apr 3;12:4. doi: 10.1186/s40694-025-00194-9 (PMC11969743; doi:10.1186/s40694-025-00194-9)

**Additional file 5.** RT-PCR (gel electrophoresis) product specificity checks for *18S, eriE, eriG, eriI, eriC, eriJ, eriB,* and *eriM* RT-qPCR primers for all working stock cDNA dilutions. See Methods regarding the *eriI* check in fruiting body samples.


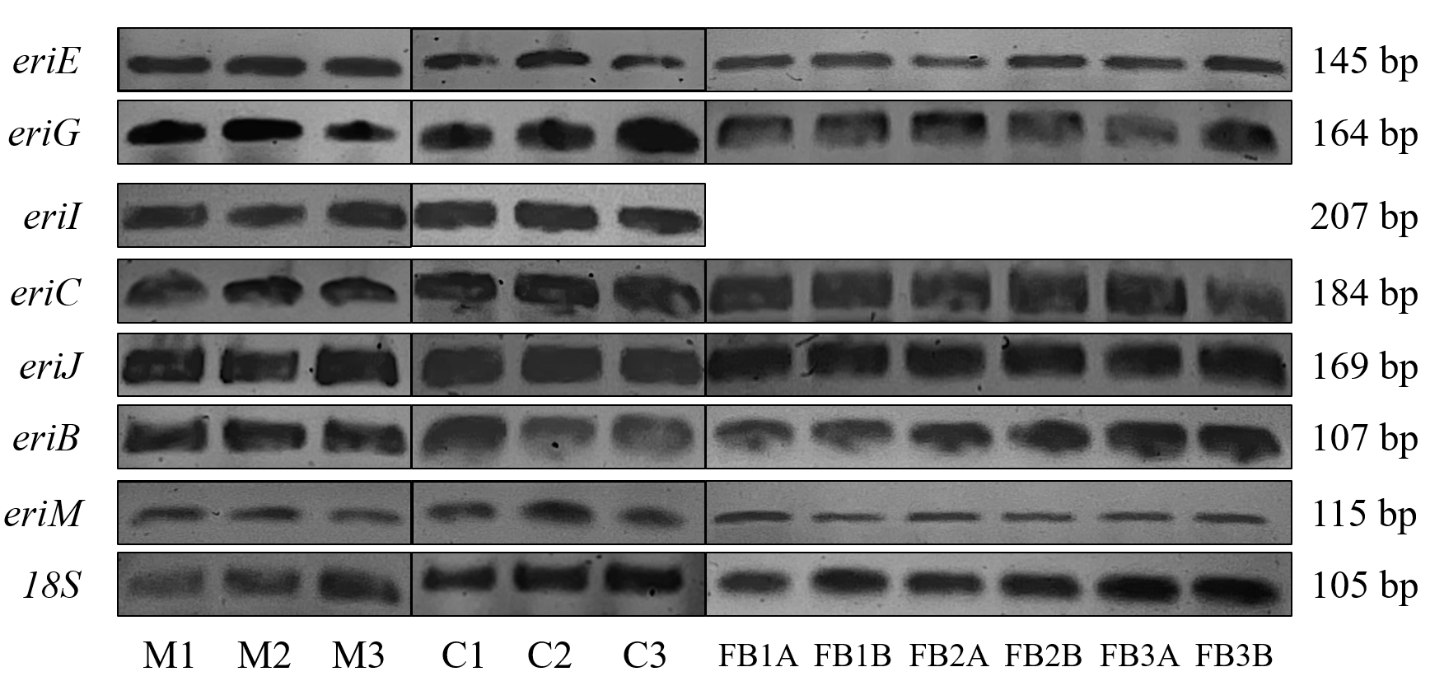

Supplement: Supplementary file 5 — Additional file 5. Gel electrophoresis specificity checks for RT-qPCR primers. See Methods regarding the eriI check in fruit body samples. [file 40694_2025_194_MOESM5_ESM.docx]
