# Supplementary material for: Influences of substrate and tissue type on erinacine production and biosynthetic gene expression in Hericium erinaceus
Source: Fungal Biol Biotechnol. 2025 Apr 3;12:4. doi: 10.1186/s40694-025-00194-9 (PMC11969743; doi:10.1186/s40694-025-00194-9)
Supplement: Supplementary file 6 — Additional file 6. UV-Vis and mass spectral signatures for erinacines Q, P, A, and C [file 40694_2025_194_MOESM6_ESM.docx]

**Additional file 6.** UV-Vis and mass spectral signatures for erinacines Q, P, A, and C as determined by Agilent 1260 Infinity II HPLC-DAD (Fungi Perfecti, LLC) and Agilent 6130 Single Quadrupole LC-MS (EZ Labs, LLC), respectively.
